# “*A Lot of People Are Struggling Privately. They Don’t Know Where to Go or They’re Not Sure of What to Do*”: Frontline Service Provider Perspectives of the Nature of Household Food Insecurity in Scotland

**DOI:** 10.3390/ijerph15122738

**Published:** 2018-12-04

**Authors:** Flora Douglas, Fiona MacKenzie, Ourega-Zoé Ejebu, Stephen Whybrow, Ada L. Garcia, Lynda McKenzie, Anne Ludbrook, Elizabeth Dowler

**Affiliations:** 1 School of Nursing and Midwifery, Robert Gordon University, Aberdeen AB10 7QG, Scotland; 2 Institute of Applied Health Sciences, University of Aberdeen, Aberdeen AB25 2ZD, Scotland; famackenzie68@gmail.com; 3 Health Economics Research Unit, University of Aberdeen, Aberdeen AB25 2ZD, Scotland; oejebu@abdn.ac.uk (O.-Z.E.); l.mckenzie@abdn.ac.uk (L.M.); a.ludbrook@abdn.ac.uk (A.L.); 4 The Rowett Institute, University of Aberdeen, Aberdeen AB25 2ZD, Scotland; stephen.whybrow@abdn.ac.uk; 5 Human Nutrition, School of Medicine, Dentistry and Nursing, College of Medical, Veterinary and Life Sciences, University of Glasgow, Glasgow G31 2ER, Scotland; Ada.Garcia@glasgow.ac.uk; 6 Emeritus Professor of Food & Social Policy, Department Sociology, University of Warwick, Coventry CV4 7AL, UK; Elizabeth.Dowler@warwick.ac.uk

**Keywords:** household food insecurity, food poverty, Scotland, low income, families, children, women, older people, qualitative

## Abstract

This qualitative study explored frontline service providers’ perceptions of the nature of food insecurity in Scotland in 2015 to inform national policy and the provision of locally-based support for ‘at risk’ groups. A country-wide in-depth interview study was undertaken with informants from 25 health, social care, and third sector organisations. The study investigated informants’ perspectives associated with how food insecurity was manifesting itself locally, and what was happening at the local level in response to the existence of food insecurity. Data analysis revealed three key themes. First, the *multiple faces and factors of food insecurity* involving not only increased concern for previously recognised ‘at risk of food insecurity’ groups, but also similar concern held about newly food insecure groups including working families, young people and women. Secondly, respondents witnessed *stoicism and struggle*, but also resistance amongst some food insecure individuals to external offers of help. The final theme identified community *participation yet pessimism* associated with addressing current and future needs of food insecure groups. These findings have important implications for the design and delivery of health and social policy in Scotland and other countries facing similar challenges.

## 1. Introduction and Background

Household food insecurity has re-emerged as a subject of public health and social policy, civic and political concern in Scotland and the rest of the UK [1,2,3,4,5,6,7,8]. Household food insecurity is the experience associated with “*the inability to acquire or consume an adequate quality or sufficient quantity of food in socially acceptable ways, or the uncertainty that one will be able to do so*” [9]. Globally, household food insecurity is recognized as a problem in low income households in high income countries [10,11,12,13]. Household food insecurity prevalence data are not routinely captured and monitored in the UK [14] but it was estimated in 2014, in a one-off UN global survey, that 10.1% of the UK population were food insecure to some degree [15]. Despite the small sample size (1000 adults for the whole of the UK), this indicated the existence of a significant and real problem [16]. However, in the absence of food insecurity prevalence data, much of the current concern about this issue in the UK was triggered by the emergence of increased numbers, and greater visibility, of charitable emergency food assistance programmes (so-called food banks), which followed the UK economic crisis and reduced government spending in its aftermath [1,2,3,4,5,6,7,8]. The numbers of people seeking help from such sources has reached unprecedented levels since the mid-2000s [17], with the causes being politically disputed [7].

At the same time, within the Scottish context, a wide range of organisations and groups that had started to provide such support in their local communities expressed significant concern about the efficacy of food banks, both as a means of addressing household food insecurity and as a social justice issue [18].

Scotland is one of the four countries which makes up the United Kingdom, and operates with its own national government (within this context) which has responsibility over some devolved matters such as health care and education.

In North America, where it has been possible to compare routinely collected household food insecurity population survey data with national food bank use figures, food bank data are known to significantly underestimate population food insecurity prevalence. Twelve to fourteen percent of the Canadian population have reported some degree of food insecurity on an annual basis since 2005, yet only 20–30% of this food insecure group also reported using a food bank in the previous year [10,19]. Furthermore, it is well established that the capacity and capability of food banks to respond to growing demand for food assistance from low income communities is severely limited due to their dependence on corporate and public donations and volunteer labour [20,21]. These resources are quickly exhausted unless food is rationed or restricted and, because of the precarious and unpredictable nature of the food and volunteer labour supply, it is thought that many people who might benefit from food bank offerings do not get access to them, and therefore do not appear in food bank statistics [10,22,23]. This is of course in addition to those who might be missing from those figures through their active avoidance of this support due to shame and fear of stigma.

Scotland has a long running public health and social policy focus concerned with addressing health inequalities. This has been underpinned by an often explicit acknowledgement that life circumstances and socio-economic deprivation are primary drivers of those inequalities, and public services, including health and social care services, have been developed and delivered accordingly [24]. Population differences in self-reported dietary quality between the most and least deprived groups (as one specific domain of the experience of food insecurity) have also come under close scrutiny over some decades, related to the goal of addressing health inequalities [25]. The Scottish Diet Action Plan, (published in 1986) triggered a programme of recurring government funding over three decades, which is intended to enable low income families and neighbourhoods to gain access to affordable fruit and vegetables via local community food programmes. Typically, these include low cost food retailing outlets, budgeting and cooking skills training programmes, and in some cases, community food growing programmes [26]. It is important to note that these programmes were not set up to provide free food assistance.

### Main Study Aim

These specific concerns about the lack of valid household food insecurity data and the possible under estimation of the magnitude of the problem through use of food bank data in its absence, combined with anxieties expressed about the efficacy and sustainability of community-based food assistance programmes in dealing with the issue, resulted in a national mixed methods study being commissioned to develop a better understanding of the nature and prevalence of household food in Scotland [27]. The study was commissioned by NHS Health Scotland, the national health promotion agency, and the Scottish Government’s Rural Affairs and Environment Strategic Research programme with the aim of informing national policy and local practice.

This paper reports on the qualitative study component of the larger formative study [23], which set out to capture the perspectives and experiences of social, health and third sector practitioners, whose main role was concerned with supporting economically and socially vulnerable groups. These groups were considered to be key informants likely to have frontline, locally based experience and knowledge of that wider picture of household food insecurity within their respective communities, through their day-to-day engagement with people requiring their input because of food insecurity, but who may not necessarily be engaging with food assistance programmes. Some of the third sector practitioners were drawn from some of those long standing community food programmes described above. This work was commissioned to complement other research that was underway at the same time that was focused on capturing the perspectives of those with direct lived experience of food insecurity.

## 2. Materials and Methods

This was a qualitative research study informed by Grounded Theory (GT) principles and techniques [28]. The decision to use GT principles as the research framework within which to identify participants, generate, analyse and think about the data was largely pragmatic, i.e., it offered a conceptually congruent set of guidelines and principles to guide the research [29,30]. As discussed above, the study objectives had been developed on the basis of emergent concerns expressed by the policy, practitioner and civic society communities within Scotland. Consequently, an interview study was undertaken with community-based health, social care and third sector staff who were concerned with the care and support of so-called vulnerable groups. The sampling frame was discussed and agreed with the research commissioners as the study progressed. Older people and those who were, or were at risk of being, destitute (e.g., homeless groups, travelling people, asylum seekers) were of particular interest to the research commissioners. The rationale for participant selection was based on identifying professionals and practitioners who had primary responsibility for some aspect of health or social care at the individual and the community level, for groups considered to be economically and socially vulnerable and, importantly, had been operating in this role for some time, preferably prior to the aforementioned economic crisis. The research commissioners were also keen to capture the perspectives and experiences of those practitioners working within the community food programmes (as described above) who were perceived to have relevant local knowledge and experience of working alongside communities affected by varying degrees of economic deprivation and vulnerability, which was known to present a significant challenge in their ability to access to healthy foods.

Two interview topic guides were developed to guide the discussions and to enable the researchers to combine inductive and deductive reasoning to generate and analyse the data. The guides themselves were generated based upon the main study objectives and a series of iterative discussions between the research commissioners and the research team. Topics in the interview guides included participants’ views about:

- what it means to live in household food insecurity in Scotland;
- which population groups were considered most affected by food insecurity;
- the main drivers of household food insecurity
- community responses to those trends and;
- notions of effective intervention/policy changes required to reduce the numbers of people seeking help with feeding.

Informants from community food programmes were also asked about how their organisation was alleviating food poverty at the current time, what they thought they might be doing to alleviate food insecurity in their community in the future, and their ideas or views about alternative models, or means required to address food insecurity.

The study was based on the conceptual definition of household food insecurity, which recognises the experience of food insecurity as one that negatively impacts nutritional and psycho-social domains of human existence, i.e., “*the inability to acquire or consume an adequate quality or sufficient quantity of food in socially acceptable ways, or the uncertainty that one will be able to do so*” [9].

The study protocol and associated materials were reviewed and endorsed by the University of Aberdeen’s Rowett Research Institute’s Human Studies Ethical Review PanelProject Review No. 2015-Douglas-01. The manuscript was written in accordance with the RATS qualitative research review guidelines [31].

A combination of purposive and maximum variation sampling was used to recruit informants to the study. As a national study, it was important to try to capture views from the different types of professional groups of interest across the whole country. Therefore we sought to engage with individuals from the different professional groups in urban, remote and rural contexts, the length and breadth of the country.

The majority of interviews took place by phone and lasted between 30 min to an hour. Two researchers (F.D. and F.McK.) undertook the interviews, and data collection stopped at the point that it became clear no new data was emerging from the interviews. All interviews were audio recorded and transcribed verbatim with informants’ consent.

Data were analysed using a thematic content analysis approach. This method is commonly used for health-related research and is particularly useful for exploring questions about meaningful issues amongst a particular study group of interest [30,32]. The basis of this approach is to reduce the multiple individual responses and identify common patterns or themes in the data, as well as so-called ‘deviant case’ issues. At the initial stage of the analysis, a sample of interview transcripts was read and re-read independently by two researchers to identify the key concepts and themes, and a draft coding index was drawn up. The researchers met to discuss their initial analysis: areas of difference were identified and where different ideas about what particular instances of the interview discourse represented, these were discussed and agreed. The final version of the thematic index was also agreed through discussion, and all transcripts were coded manually. Memos and notes of emerging themes, issues and patterns were also recorded during this process and were referred to during the analysis. Constant comparison method was used throughout to confirm coding consistency and assignation of coded data to the emergent themes and categories, and to check that possible new themes were not being overlooked. Every attempt was made to search for disconfirming data within the data set. Data were also scrutinised for the possibility of dominant and/or marginalised viewpoints.

## 3. Results

Ten informants representing community food programmes and 15 informants from organisations concerned with the care and support of vulnerable groups were recruited to the study. The combined sample of informants was drawn from across Scotland and represented some of the key organisations and services that were being delivered in diverse urban, rural and remote locations. (see Appendix A for a detailed breakdown of the study participants’ characteristics). The community food programme informants were people who worked in programmes that were offering multiple services, including low cost food retailing, and/or training and development programmes and/or community growing and gardening schemes. This group also contained three NHS-employed community food development staff. Although not originally set up for this purpose, six community food programmes also begun offering a take-home free food parcels (i.e., similar to a food bank service). One informant representing a recently opened food bank also took part. The 15 health, social care, and third sector participants, were drawn from a range of community-based care and support services agencies. It is important to note that those interviewed were also targeted on the basis of having been in their current post over a number of years so that they could provide insights from practice about the current position compared to their pre-recession experience.

Three major themes that emerged from this analysis are discussed in this paper, i.e., the *faces and factors of food insecurity* in Scotland associated with emergent food insecure groups, and those groups previously recognised to be at risk; *stoicism and struggle* witnessed at the individual level amongst people affected by food insecurity, and the community *participation yet pessimism* that surfaced in relation to the challenge of responding to expressed local feeding needs now and into the future.

### 3.1. Faces and Factors of Food Insecurity

Two fundamental issues explored at the beginning of each interview were informants’ perspectives about who they believed to be, and encountered to be, most obviously affected by household food insecurity and what they believed was causing their food insecurity. The most common responses that surfaced here were not only more concern and anxiety for groups previously well known to them but also great concern for groups they had never previously considered to be affected by food insecurity.

Families with young children, young people and women were identified as emergent groups and sections of particular concern. This anxiety is illustrated by the following quotes from two different development workers based in urban locations in the north and central parts of Scotland:

*I’ve got families that the parents do without, so that the child has got what they need to have, and it means that society is becoming even more uneven than it used to be before*. (Development Worker, urban),

and:

*You’ve got people making choices about the kids clothing and shoes or a meal, you’ve got adults, women in particular I suspect, not eating properly so the kids are fed*. (Development Worker, urban).

While both quotes typify concerns for parents and children in general, the second quote illustrates the particular concern for women with children, some of whom were believed to be sacrificing their own food resources to ensure their children could eat. Some reported specific concerns about pregnant women.

The notion that families with young children were more obviously affected by food insecurity now compared to the past was linked to their having insufficient household incomes. Much of the public discourse about the rise of food banks in the UK has been linked to changes in UK government policy and related social security entitlements that were associated with unemployment (job seekers) or sickness or disability benefits. Yet many of our informants described supporting or encountering people who were working but not earning enough to cover their necessary household bills, illustrated here:

*We have families, I have a lot of experience with people who work very hard and work long hours, to support their families, and still at the end of the week don’t have enough money for basic food* (Rural, voluntary org, family worker).

Not only were people described as living on low incomes from their employment, but the issue of unpredictable levels of income was also flagged as an underlying determinant of the food insecurity. In this next illustrative quote, this welfare support worker who was based in a rural community in mid-east Scotland talks about her frustration that her clients were very keen to find work but were unable to survive on the hourly rates and number of hours on offer from local employers:

*… one of the bigger employers in this area is a market gardener, who employs people through agencies, very often on short term contracts. They’ll be zero hour contracts and certainly, because it’s off season at the moment, a lot of people get signed off or maybe only get one shift a week, so they may be in employment, however, their income is so low that they actually can’t pay their bills*. (Welfare support, rural).

Indeed, it was notable that local food production and food processing work featured in other rural participants’ accounts as examples of industries which offered very low and unpredictable levels of pay for their workers.

However, picking up on the experiences of some other community-based development workers, we also found degrees of frustration expressed about recent policy changes to UK social security entitlement, which was perceived to be driving people into destitution as their benefits were reduced or removed for periods of time. These changes were viewed by many as a primary cause of household food insecurity: an argument typified in this quote:

*He said that his benefits had changed, and he’d had to make a new claim or something, and there was a delay in getting his benefits. And this is often what we’re told; that people have a delay, they’ve got to make a new claim, they get less money than they think they would get, they’ve got to wait an extra week or a fortnight to get the money. And in the meantime they often don’t have anything, and they don’t have any fall back*. (Social worker, island).

A few participants also talked about policy changes that were counterproductive to the aim of getting people off government support and into paid employment. These quotes from a community-based nurse located in a remote island community, and an urban-based development worker illustrate this notion of people cycling back into debt and poverty as they tried to move into paid work and off social security:

*Younger people, who are of working age, have a much more variable source of income. If they’re in employment that’s fine; they might be in low employment and things are difficult. If they’re moving in and out of employment, and in and out of the benefits system, it seems to me that it’s very precarious* (Nurse, rural/remote).

and

*The problem is that when they start work their first pay day may not be for four weeks. They then have got to work out how they’re going to survive for that period. For many of them, the only solution is actually getting into debt of some kind. There’s meant to be all kind of safety nets around that but that’s just not happening in practical terms* (Development worker, urban).

These quotes also highlight the common concern expressed by our informants about younger people being amongst those new groups perceived to be most badly affected by and at risk of household food insecurity. In the next quote, the same community-based nurse, cited above, raises the issue of their existing vulnerability as an economically disadvantaged group being exacerbated by poor social support, in this case, emanating from people having to move away from the island to find work:

*There could be any, they’re working age people, and I would say that it’s more typical for the younger end of working age, but it could be older people, in the working age group, who’ve had some other life crisis, like their family has broken up. They’ve had to move away from their family and from the way that they used to do things*. (Nurse, remote island).

During the time period in which the interviews took place there had also been an economic downturn in some industries in Scotland, including the oil and gas energy sector. This was linked to the experience reported by a few urban-based informants of having dealt with or being aware that previously high income earners were struggling as a consequence of losing their jobs and not being able to feed themselves, despite having a lot of expensive possessions, highlighted by this quote:

*I’ve actually had people coming in with the best cars, the best fancy phones and whatever - not a lot, but I have had, coming in with all the flashiest of stuff saying that they’ve got a problem. The problem is they can’t pay their bills. It doesn’t matter that their bills are ten times higher than maybe somebody else’s bills, they still come to the end of the month and they can’t pay, you know? … So it’s kind of like hidden, I suppose. It’s not what you expect*. (Community Food Programme Development, urban).

This quote (above) also touches on an issue that was remarked upon by many of the informants (regardless of income status) in terms of the ‘hidden’ nature of food insecurity; a theme that is picked up again later in the paper.

The situation for older people which was of initial, primary concern from the research commissioner’s perspective, was more nuanced. Generally speaking, this study found most informants expressed less concern about older than younger people. This was often described in terms of an acknowledgement that while many older people lived on a low income, it was a predictable and relatively stable income that people had learned to live within and budget accordingly. In addition, older people were viewed as possessing all the necessary additional resources need to provide a constant, if limited, food and meal supply in the home, i.e., had the necessary food preparation, storage and cooking equipment that had been accumulated over their lifetimes. Older people were considered better able to cope with household food insecurity as illustrated here:

*… we very seldom have to help them [older people] out with money or with food*. (Nurse, rural/remote).

Mention was also made of older people not appearing at food banks in great numbers, which was interpreted by some to mean they were not in need of help. Interestingly, this was something that the research commissioners had noted, but had viewed as an indication of there being a problem, not a reassurance that all was well.

However, there were a minority of informants who were working directly with older people in their homes who were concerned about what they were seeing in practice (e.g., noticing that their clients’ cupboards and fridges had little or no food during home visits) that led them to believe that some of their older clients were food insecure. These older people were also commonly described as denying they were having a problem with this and commonly refused offers of a referral to a food bank. Older carers were also highlighted as a group of concern.

However, even groups normally in regular contact with health and social care services were reported as being more badly affected by food poverty compared to the past, illustrated thus:

*Definitely an increase in people who are long term sick who’d sort of settled down to a lifestyle where they understood their income so you may have had somebody who for 15 years had been in receipt of a benefit that was related to their ill-health who found themselves unchallenged around that, their rent was being paid, their council tax was being paid and they understood how much they had to live on every week*. (Development worker, urban).

This quote also illustrates a commonly cited participant observation that financial instability and unpredictability appeared to have become the norm for many people who were in receipt of social security payments due to long term ill health, and which was perceived to have occurred as a consequence of changes to government policy. Those changes to the previous pattern of timing and level of payment had made household income difficult to manage as a result.

These perceptions about the prevalence of insufficient and unpredictable income in Scottish households fit with informants’ views about what it means to be food insecure in Scotland; i.e., lacking choice and being compelled to seek out cheap, nutrient poor food to survive, illustrated in this quote from an urban-based community food initiative informant:

*I suppose the general idea is that you don’t have enough food to eat but my thinking is, it’s not the right food, not nutritious food that people can’t afford. Or they’re making choices out of necessity as to what’s available rather than what they would probably like to eat. As you know, a lot of people—you see it in the supermarkets when they’re reducing the food there are people queuing up just waiting for the food to be reduced*. (Community food programme informant, mixed/urban rural).

### 3.2. Stoicism and Struggle

It is important to stress that this study was concerned with community caregivers or support workers views’ of their clients’ food security status and that we were not able to explore their clients’ perspectives directly during this particular study. However, this research was concerned to understand how those care givers were drawing conclusions about their clients, and what evidence they used to conclude that individuals were dealing with food insecurity. We found informants were using a wide range of different information sources, including perceptions of their clients’ behaviours and attitudes, and assessments about their physical appearance, as well as their dialogue with them. It was from these data that the theme of widespread individual (and private) *struggle and stoicism* emerged.

The behaviours and attitudes participants cited ranged from actions intended to keep up appearances of being able to manage, to denial of there being a problem in the household, through to overt refusal of food bank referrals. This notion of private and long-term struggle is illustrated by this quote from a community nurse who was working in a part of the country where large numbers of long term unemployed people live:

.., *a lot of people are struggling privately. They don’t know where to go or they’re not sure of what to do, you know, or they’ve been sanctioned. Can you appeal this, can you, you know, do different things about that, and they’re struggling day to day. “Oh today I’ve got some money, tomorrow I don’t have anything.” They’ll not worry about tomorrow, because they’re managing with today; that kind of idea* (Outreach community nurse, mixed urban/rural).

A few informants talked about seeing people they had been dealing with over time looking progressively unwell and noticing or being concerned about their clients’ appearance, the lack of food they observed in some of their clients’ homes, and noticing that basic household furniture and fittings were missing from their houses (presumably sold to raise money for food), as things that led them to believe that some people were struggling with food poverty, illustrated thus:

*You know on a couple of occasions we have seen people come in who are clearly you know, look unwell and you know are struggling*, (Housing Regeneration Manager, urban).

These discussions of private struggles were also underpinned by notions of underlying pride that, from the perspective of the interview participants, prevented people seeking help, highlighted here:

*I think for people that are too proud to come forward … you know, older people who worked all their lives, who don’t expect to find themselves in the kind of poverty that they find themselves in* (Manager Counselling Charity, urban).

Many also talked about people they considered to be food insecure being consumed with embarrassment during conversations the informant had initiated that were intended to help, illustrated thus:

*The number of people that have been referred to myself that have been working and they have described financial hardship for a number of reasons and I’ve offered to make these referrals to the food banks, whichever one is more accessible for them, and they really just become very embarrassed. And then when I probe just that wee bit further about how they’re going to provide for their families and themselves they kind of say that they’re going to rely on their families and friends to do that*. (Community link worker, urban).

Perhaps another reflection of the widespread private stoicism described above was the finding that participants who were involved with the delivery or management of community food programmes had noticed increased recent uptake of, and interest in, any food and budgeting training and cooking skills development courses. It seems that this had happened ‘organically’ as there had been no significant increase in their promotion and marketing of those courses. Yet people were signing up to them. They also noticed increased demand for their low cost food retailing services (fruit and vegetables). Moreover, a few had noticed more people growing their own food in community gardens and allotments, and that demand for community growing spaces was increasing. This was interpreted by some to mean that people were taking active, self–initiated steps to mitigate their situation.

Conversely, a few participants reported finding some people were more willing and able to ask for help, and/or accepting of their referrals to food banks to help them acquire food, compared to their previous experience. This effect was theorised to have occurred because they believed emergency food aid centres, such as food banks, were more commonly known and talked about compared to the past. In effect, using a food bank had become more socially acceptable making it easier for some people to accept this type of help when offered.

### 3.3. Participation Yet Pessimism

To reiterate, we deliberately set out to engage with professionals and third sector workers who had long term experience of supporting groups who were considered to be vulnerable due to their economic or social circumstances, or had health care needs, or who had long standing and established experience of designing and running community-based food programmes intended to enable low income households to purchase, prepare and consume healthy foods. Yet we found that both groups had in-depth knowledge and experience of the role and operation of food banks within their local communities. In exploring responses to food insecurity at the community level, the overriding theme to emerge was that the community had actively *participated* in attempting to support local people in food crisis, but was *pessimistic* and sceptical about its effectiveness as a solution now or in the future.

All the community food programmes that we engaged with for this study had been operating for over a decade prior to this study, without a food bank, and all reported a very similar experience in relation to dealing with local requests for help with feeding. All had added a food bank operation to their range of programmes or services in recent months in response to those appeals. Those appeals appear to have come from two groups: health and social care professionals who had lobbied them for emergency food parcels on behalf of patients or clients they believed were in food crisis; and direct requests from local people in food crisis, who knew of their previous existence as a local, low-cost food programme.

Yet there were mixed views amongst community food programme informants about the role of food banks and the impact that they had in addressing household food insecurity in Scotland. Overriding participants’ narratives about the community responses were notions of pessimism, scepticism and concern about the role and efficacy of food banks as a solution to household food insecurity. This next illustrative quote highlights the dilemma expressed by many of the deep concern they had for members of the local community who were perceived to be suffering, feeling the need to help them, but at the same time being aware that a food bank response did not address the problem:

*I feel outrage that people have to go through this kind of terrible suffering, food poverty, in this age! And, you know, I’m sure there are many people kind of saying the same thing. You know, I’m very satisfied that I’ve got this kind of work, where I feel I can make a difference now and again, but I’m also overwhelmed by the fact that I know that’s just almost a drop in the ocean. There are many, many people that need help and support* (Welfare support assistant, mixed urban rural).

The sense of anger expressed in this quote was also apparent in other community food informants’ accounts. This anger and frustration was centred not only on the individual suffering witnessed day-to-day but was also focussed on their organisations feeling obliged to help and becoming a de facto social safety net as a consequence. This next quote highlights some resentment directed towards mainstream (government-funded) organisations and agencies about expectations that were perceived to have been placed on poorly-resourced charities to help alleviate local suffering:

*Well, I think it’s one of the few areas that I’m aware of that the only response is, “Go to the voluntary sector.” I can’t think of very many other services that are related to similar sorts of outcomes, where the response is, “Go to a food bank, go to the voluntary sector,” especially food banks, who get very little money from anywhere. I think part of the problem is that it’s a free service that’s been offered that we may have to challenge, in the future. I can’t think of anything else, in a similar situation, where people say, “Go to your local church, they’ll help you out,” which is what people are, in effect, saying about food banks, you know, main stream services, main stream agencies, Local Authority’s and whoever else. I find that very, very worrying* (Community food programme informant, mixed urban).

In addition, there was an overriding sense of pessimism amongst informants that this picture and these trends in household food insecurity were about to change in the short-term. Most believed it was likely to become worse rather than better, particularly when the additional proposed changes to the social security system were enacted in the near future. This proposed change referred to here is the introduction of new UK Government policy associated with the so-called Universal Credit system of social security payment that would see the scrapping of fortnightly payment of separate types of security payments such e.g. family tax credits, housing benefit unemployment benefit etc. in favour of a single, monthly payment system. This fear and pessimism is illustrated here:

*And I don’t know what would happen to the other half; I’m really frightened, and because, as I say, we have designed and promoted ourselves as a place of last resort for funders, you know, it’s the only option available; the last option available. I don’t know what would happen* [to them]. (Community Food Program, urban).

Some informants predicted future expansion of the food bank service on the basis that there would be an ongoing need to support hungry people, highlighted in this quote:

*I think food poverty in Scotland isn’t something that’s going to be resolved overnight. I think it’s going to be a long … there’s going to need to be looking at like longer term more sustainable solutions but I feel that until these things are achieved, food banks are now kind of part of the dialogue and will be for, maybe longer term, until adaptions are made to the welfare system and especially with regard to sanctions. But as a result of that, food banks will be … will have a kind of longer-term role to play* (Community food programme, urban).

Almost all the community food programme informants indicated they did not believe that food banks were a positive development or an effective or sustainable solution, but could not envisage them becoming redundant soon. Some talked about wishing to develop a different ‘model’ of local assistance, in the future, that would enable people to buy low-cost, healthy food according to their individual dietary needs and preferences, as opposed to being handed a free food parcel. All community food programme informants talked about trying to ensure that their clients had access to as nutritious a food parcel as they were able to supply. Most also commented at some point in their interview about the limited nature (in terms of nutritional quality and dietary preference) and lack of choice their clients had in the food were given. In the context of these discussions, it was also interesting to note that a few informants also talked about the unpredictable and limited nature of the food supply available to them (sourced from public and corporate donations, and allocations from franchised food surplus distributors) and the challenge they had in meeting the needs of their local community. This next quote illustrates the limited and unhealthy nature of the food informants received from a national food surplus redistribution operation:

*The council gave us money to join (food surplus distributor), but as to date since we started with (food surplus distributor), the amount of produce we’ve been able to use is less than 20 kg a week. Because we can’t take chilled produce, we can’t take frozen produce, so the ambient temperature … they’ve given us a lot more than that but the biggest item by weight we’ve had has been diet Irn-Bru (soft drink). I would actually use that as an example of something with no nutritional value whatsoever. The second biggest item we’ve had, not by weight but by quantity, has been salt and vinegar crisps* (CFI mixed urban/rural).

One social care informant, working with young people at risk of homelessness, described his frustration associated with observing that the components of food bank parcels did not necessarily match the healthy eating on a budget training that his young clients were being directed towards:

*Well, we’ve tried to work with food banks. We haven’t always found that entirely easy to be honest. We’ve produced a range of recipes and that sort of thing aimed at healthy eating on a budget and not all of the food banks are providing a great balance of food within the food boxes and that’s not in any way to throw any blame at them, they can only allocate what they get, but access to fresh food and things can be quite difficult. We’ve done a range of training workshops with young people primarily aimed at teaching people how to create meals that don’t require cooking but it’s hard to get that matched up well with the contents of the food boxes*. (Homeless organisation, urban).

Moreover, it also became apparent when talking to the community food programme informants that food banks were not well placed to meet the needs of people with a long term health condition or conditions seeking help from them.

Whilst food banks were predicted to remain a response to the existence of hungry people in local communities, overall, informants believed it was action to increase the levels and predictably of people’s income that would make the biggest impact on food insecurity in Scotland. Support for young people to get into employment and get access to decent housing were also high on the list of things informants mentioned here. While a few described their aspirations that locally grown food would be part of the answer, they expressed disbelief that it ever would be for those on very low incomes, as it was considered well out of reach, cost wise, for those people.

## 4. Discussion

While this study revealed observations and concerns about groups of people historically well known to services due to their economic and social vulnerability, or frank destitution, this study also revealed widespread perceptions and concern about groups (particularly) families with young children and young people never previously considered to have been so obviously affected by food insecurity in affluent contexts. Income insufficiency was thought to be the primary cause of this by the majority of our study participants. This was related to the nature of work i.e., low wages, and insufficient and unpredictable hours of work, as well as changes to social security entitlement changes that previously boosted the take-home pay of those in low wage employment. While concepts of food insecurity and those affected by it, described in this study, did include descriptions of destitute life circumstances and/or crisis situations involving obvious hunger, those accounts also included concern for a growing number of people living within communities who were perceived to be dealing with food insecurity but were not necessarily totally insolvent or going hungry per se. This was characterized by our study participants as something they associated with having to eat (or survive) on cheap, poor quality foods due to having insufficient household income. Concerns about groups and households not obviously experiencing destitution but who are still considered to be at risk of food insecurity, due to inadequate income, has also been highlighted elsewhere [33,34].

The UK’s Joseph Rowntree Foundation, for example, has found that those living in the lowest income decile households in the UK are routinely spending 20–23% of their household income on food and non-alcoholic beverages compared to 11% allocated to this expenditure by those living in average to above average income households [35]. Our quantitative study found those living in households with below 60% average income were routinely spending between 18–23% on food and non-alcoholic beverages in Scotland between 2005 and 2012 [27]. Caraher and Furey also estimated that the lowest income decile group in the UK would have to spend 40% of their household income if they were to buy what they describe as a consensually healthy diet in the year ending 2016/2017 [16]. Furthermore, the cost of purchasing healthy food and beverages (compared to unhealthy foods) has been steadily been rising in the UK since 2002, and predictions that healthy diets will become less affordable over time suggests, as Jones et al. also argue, that there are significant implications for individual food security and population health going forward [36]. It should perhaps be no surprise therefore, that low income households in Scotland are consistently failing to achieve population healthy eating targets in Scotland [37].

In addition, while hunger due to destitution is no less a public health and social concern in its own right in the UK and elsewhere, food insecurity without hunger in high income countries (like Scotland) is increasingly understood to be the more common experience, but also as damaging to health [33,38,39]. Recent UK research indicates that the numbers of people who report skipping meals in the previous year due to economic constraints had risen from 13% in 1983 to 28% in 2012 [40]. Our participants’ observations and experiences of encountering an increasing number of people and groups of people affected by food insecurity, characterized by many of them to be synonymous in the Scottish context with having to eat cheap, nutrient poor food to deal with household income insufficiency, and is also consistent with this finding.

These findings therefore have important implications for public health and social policy makers, researchers and practitioners concerned with population health improvement and promotion, who are facing growing health care costs associated with non-communicable diseases. There is growing, evidence-based recognition that chronic compromises in dietary quality (due to the experience of food insecurity), rather than periodic episodes of hunger, are not only the more common experience of food insecurity in high income countries like Scotland, but also the more likely cause of a wide range of negative physical and mental health outcomes [41]. Food insecurity is known to increase the risk of a range of chronic, non-communicable health conditions and mental health related problems such as depression [42,43,44]. It is also notable that Scotland ranks second behind the US (in the top 20 OECD countries) in terms of the population prevalence of overweight and obesity. The Scottish Public Health Observatory has estimated that a fifth of all cases of obesity here can be attributed to living in deprivation [45]. Living in poverty in high income countries increases the risk for overweight and obesity [46], and while the mechanisms behind this are not fully understood, it is suggested that those living in poverty consume large quantities of highly energy dense foods (and therefore excessive calories) due to their appealing combination of affordability and palatability [45,47]. Therefore it maybe that policy interventions aimed at maximizing household income, such as those currently being tested in the Scottish context associated with addressing child poverty [48] and a universal basic income [49], hold more promise in addressing the root causes and health consequences of food insecurity, compared with an emergency food-based policy response.

It is also important to remember that those living in destitution (and their food security status) remain a significant public health concern in the UK [17,50], and those interviewed for this study were acutely aware of this, regularly highlighting their observations of increased hardship and concern about the significant challenges faced by those groups they were more commonly used to dealing with in their work. This increase in adversity was frequently linked to changes to social security entitlements associated with unemployment and sickness benefits due to changes in UK government policy [48,51].

In relation to older people, the target group of concern at the outset of this research, the mixed picture that emerged here reinforces the need for vigilance and continued close monitoring of this group in relation to food insecurity. Whilst pensioner poverty has declined in Scotland in recent times [51] and some older people were perceived and observed to be living without obvious barriers to food resources, from our study participants’ perspectives there was still cause for concern. That older people are not turning up at food banks in the same numbers of younger people is not an indication that all is well with this group.

Stoic struggle, and resistance to the notion of being thought of as being unable to feed oneself, was featured amongst accounts of encounters with older people, and other groups too. This study found that professional offers of referrals to food banks were being turned down by some who had given such cause for concern. This resistance to being considered or revealed as being incapable of feeding oneself should be no surprise when considering Poppendieck’s and Chilton’s arguments that, relying on charity (to feed oneself) not only undermines basic human dignity, but also risks drawing public attention to one’s reduced capability as family providers, protectors and consumers [22,52]. In consumer societies like the UK, this defective status represents a significant challenge to an individual’s self-worth and wellbeing [53]. Indeed, the experience of living in poverty is known to be accompanied by feelings of great shame and fear of stigma [54]. Moreover, the drive to ‘keep up appearances’ and pretence to appear ‘normal’ and ‘respectable’ is universally experienced in cultures of widely varying economic circumstances throughout the world [55]. There is certainly a growing body of experiential studies, investigating food bank users’ perspectives, that has revealed that their use is the action of last resort, and is commonly accompanied by feelings of great shame and powerlessness [56,57,58,59,60,61]. This apparently human instinct, to hide individual household food insecurity from public and professional scrutiny, has significant implications for the design and delivery of policies intended to address it. For example, vigilance needs to be maintained through research and service evaluations to reduce the risk that people and households struggling to cope with food insecurity are missing out on support they are entitled or eligible to receive, by those front line service providers who interact with them.

Whilst families with young children were considered to be amongst those groups giving cause for concern amongst our participants, a few expressed specific anxiety about the food security status of mothers and pregnant women. Women with children, living in very low income households, are believed to be at particular risk in relation to coping with food insecurity and its potential impacts on their own food intake and health [62,63,64]. Moreover, it is not uncommon for mothers to try to optimise their children’s dietary intake at the expense of their own diets [65]. Women have been found to be less likely to present at a food bank for help in the UK [7,61], but are notably more likely to report moderate or severe food insecurity in population surveys compared to men [66]. While Loopstra and Lalor (2017) reported in their recent study of UK food bank users that men were the largest group of users (39%), lone mothers with children were the next most prominent group (13%), with households with three or more children particularly prominent amongst this group and the most vulnerable to severe food insecurity [67]. Therefore, the fears expressed by our participants appear plausible.

Almost all the community food programmes we engaged with had been operating without a food bank for over a decade but reported very similar experiences in relation being compelled to add this facility to their service offerings in response to locally expressed need in recent times. Nevertheless, there were mixed views amongst study participants about the extent to which they thought their food banks were effectively addressing their clients’ needs, a finding which concurs with the lived experiences of food bank users reported elsewhere [56,57,58,59,60]. There was also some concern expressed in this study in relation to food banks not being able to meet the needs of people with a long term health condition or conditions. This is an important issue to take note of, as people with long term conditions are disproportionately represented in UK food bank use statistics. For example, the 2017 Loopstra and Lalor survey of food banks in the UK found that 63% of food bank user respondents had a health condition, and a further 5% had someone living with a health condition in the household [67]. The experience of living with food insecurity is known to adversely affect individuals’ ability to manage their health condition and achieve optimal health outcomes [68,69,70]. Furthermore, there is also an emerging trend in the UK to suggest that more people are relying on food bank parcels on a regular basis, as opposed to this being a one off, rarely-used food crisis support [56,71]. Therefore, there is a need to undertake research with people in Scotland and the rest of the UK, who are living with a long term condition or conditions and who are affected by chronic or periodic food insecurity, to develop a better understanding of these experiences and their impacts.

These findings raise important considerations for public health and health care policy addressing household food insecurity in Scotland. Whilst Scottish Government investment in feeding assistance programmes like food bank operations and children’s holiday feeding programmes, through their Fairer Food Fund [72], might make a short term difference to those subsets of the food insecure population using them, this investment will not reach the remainder who either do not access or have no access to such support.

Almost all the study food programme informants were pessimistic about any prospect of a future reduction in demand for food banks. There is certainly evidence to indicate that those predictions have been well founded [73]. Concerns were also expressed about the sustainability of some food banks in relation to local demand outstripping supply and in relation to being unable to provide healthy nutritious food in sufficient quantity and quality to supply the needs of people seeking help from them; concerns that have been highlighted elsewhere [21,22,72,74].

This study has some important limitations that need to be borne in mind. For example, the relatively small number of participants we were able to reach within the study timeframe means that it is problematic to generalize to all possible participants throughout Scotland. However, we attempted to gain the perspectives of as varied and relevant a sample of participants as possible (as described in Section 2) and argue that the findings are theoretically generalizable for two reasons. Firstly, there is a dearth of existing studies that has explored social, health care and third sector practitioner’s perspectives and experiences of these issues. Secondly, after the study was finished we found from a series of knowledge exchange events that took place throughout Scotland, that our respondents and their narratives were not atypical [75].

A further limitation is that the study was not designed to engage with people directly affected by the lived experience of food insecurity. As discussed above, some additional work was being undertaken to explore that lived experience perspective in a separate but simultaneous piece of work in Scotland. Nevertheless, there is still a significant gap in the published literature particularly with respect to those individuals and households perceived to be experiencing food insecurity and believed to be at risk of food insecurity by practitioners and professionals working in health and social care arenas, but who are choosing not to use, or are having difficulty accessing, feeding assistance programmes. This study does not purport to represent the views of people with lived experience food insecurity, but to provide important insights into the perspectives and experiences of those frontline health and social care service providers, including those third sector and community-based programmes. These individuals are more commonly and historically used to dealing with groups and individuals who are financially or socially vulnerable; for example, who are not necessarily destitute but affected by in-work poverty, and, or who have routine health care needs. To the best of our knowledge, this is the first study of its kind to represent these views, for much of the emerging, published studies focusing on household food insecurity in the UK have been directed towards the experiences and perspectives of food assistance programme providers.

Finally, it was beyond the scope of this study to measure the extent of food insecurity but the findings stress the need to monitor food insecurity. A routine population survey tool could be added to an existing survey, such as the Scottish Health Survey, as the means by which policy can be informed by a more accurate picture of the population food insecurity prevalence, particularly given the evidence presented here of there being groups and individuals thought to be in need of help with feeding, due to income insufficiency, but who are not engaging with feeding programmes that might benefit them. However, routine surveys may still fail to capture fully the experiences of hard-to-reach groups.

## 5. Conclusions

This study set out to understand the nature of food insecurity beyond food bank provider’s experiences and it revealed widespread concern for highly vulnerable groups more commonly known to be affected or at risk of destitution; and this remains a pressing public health issue. However, it also identified concern about groups, particularly families with young children, young people, women, and some older people, who were never previously considered to have been so obviously affected by food insecurity. The notion of food insecurity underpinning this view point was frontline observations of households having to survive on cheap, poor quality foods due to insufficient income; a conclusion that may explain why low income households have been consistently failing to achieve dietary targets in Scotland over some decades. The study also revealed a sense of commonplace stoic and private struggle within some food insecure households that seemed designed to avoid revealing their condition to public view. This apparently basic human instinct to try to conceal lived experiences of food insecurity from public and professional scrutiny has significant implications for the design and delivery of policies intended to address it. Furthermore, community-created and delivered food-based responses, that have been accessed by those willing and able to use them, were understood by those setting up and operating them to be insufficient and ineffective in addressing the root causes, and for those with health conditions, not well suited to meet their needs. Therefore, these findings point to some important public health, health care, and social policy implications.

Focusing on optimizing food bank operations seems unlikely to impact on the experience of food insecurity for those people who are unable or unwilling to access a food bank. Even for those who do access food banks, their operation can be viewed as alleviating the symptoms of food insecurity rather than addressing its causes. However, in Scotland, this has recently been a primary response to addressing food insecurity at the local level, through the provision of competitive grant funding to food bank operators. Policies that focus on income maximization, on the other hand, would seem to hold more promise in enabling more people to feed themselves, according to the perspective of frontline service providers interviewed in this study. In addition, we believe it is important to capture and monitor the experience of food insecurity both quantitatively through routine population surveys, as is the case already in Canada and the US, but also qualitatively through regular engagement with people with direct, lived experience of food insecurity, both those who use and don’t use food banks, through research and dialogue. Both types of data are required to develop and monitor policy interventions intended to address food insecurity and to understand the impact of any policy changes arising.

## Appendix A

**Table A1:** Service Provider Informant Details.

Type of Organisation	Role of Interviewee	Project or Service Description	Location	Population Group Served
Service Provider	Staff nurse, Vulnerable Populations Team	Health Service representative supporting vulnerable adults	Greater Glasgow & Clyde	Vulnerable adults of all ages (16 and over)
Service Provider	Pre School Educational Home Visitor. Provides support & education to parents regarding their children’s development needs	Education & children’s services	Fife	Vulnerable parents regarding their children’s development needs
Service Provider	Deputy manager of advice and information service for vulnerable groups	Supports homeless or those at risk of homelessness	Grampian	Homeless & other groups at risk of homelessness
Service Provider	Family worker supporting vulnerable families via parent and toddler groups	Supports vulnerable families	Highlands	Vulnerable families with young children
Service Provider	Principal adult social worker for vulnerable groups	Supports disabled and other vulnerable adults	Orkney	Disabled and other vulnerable adults
Service Provider	Welfare Support Assistant for unemployed people	Supports unemployed people back into work	Fife	Unemployed people
Service Provider	Community Health Improvement Advisor	Promotes healthy eating and the prevention of chronic illnesses	Grampian	Vulnerable adults of all ages (16 and over)
Service Provider	Community Links Practitioner working in Primary Care—supports all patients in GP practice	Supports vulnerable patients	Greater Glasgow & Clyde	All patients in GP practice in community
Service Provider	Manager. Supports vulnerable groups in city	Supports people back into work. Counselling services	Grampian	Vulnerable adults of all ages (16 and over)
Service provider	Adult befriending Service co-Ordinator. Supports adults who are socially isolated in community	Supports vulnerable adults in community	Orkney	All adults who are socially isolated in community
Service Provider	Assistant Chief Executive. Supports vulnerable young people	Supports young people at risk of homelessness back into employment	Highlands	Young people at risk of homelessness
Service Provider	Development officer. Supports vulnerable adults	Supports vulnerable adults in community	Grampian	Vulnerable adults of all ages (16 and over)
Service Provider	Re-generation manager. Supports vulnerable adults	Supports vulnerable adults in community	Greater Glasgow & Clyde	Vulnerable adults of all ages (16 and over)
Service Provider	Administrator. Supports vulnerable adults	Supports vulnerable adults in community	Grampian	Vulnerable adults of all ages (16 and over)
Service Provider	Integration Development worker. Supports asylum-seekers and refugees	Supports asylum-seekers and refugees	Greater Glasgow and Clyde	Asylum-seekers and refugees

**Table A2:** Community Food Programme Informant Details.

Type of Organisation	Role of Interviewee	Project or Service Description	Health Board Area	Population Group Served
Community Food Initiative: Community food programme with food bank	Manager of community food and health initiative	To improve people’s health by providing them with nutritious food and cooking and nutrition classes	Greater Glasgow & Clyde	Vulnerable adults on a low income
Community Food Initiative: Food bank only	Manager of food bank	Food bank	Greater Glasgow & Clyde	Vulnerable children and adults on a low income
Community Food Initiative: Community food programme without food bank	Project Assistant at voluntary community health project	Voluntary community project which promotes healthy eating/living	Forth Valley	Vulnerable adults on a low income
Community Food Initiative: Community food programme with food bank	Chief Executive. Supports vulnerable adults	To improve health and wellbeing and to increase employability	Grampian	Vulnerable adults on a low income
Community Food Initiative: Community food programme without food bank	Community Food Development Worker for community food and health project	Supports people at risk of homelessness, offenders or those at risk of offending	Fife	Vulnerable adults on a low income
Community Food Initiative: Community food programme without food bank	Manager of a healthy living centre	Tries to alleviate food poverty through their education and promotion work	Greater Glasgow & Clyde	Vulnerable adults on a low income
Community Food Initiative: Food bank only	Development worker at the foodbank	Promotes healthy eating in local schools and nurseries and runs cookery classes	Greater Glasgow & Clyde	Vulnerable children and adults living in community
Community Food Initiative: Community food programme with food bank	Foodbank coordinator at national voluntary organisation	Food bank and drop-in advice service	Dumfries and Galloway	Vulnerable children and adults on a low income
Community Food Initiative: Community garden	Volunteer coordinator at community food and health project	Promotes healthy eating via cookery classes and workshops. Sells cheap fruit and veg	Fife	All residents living in the local village
Community Food Initiative: Community food programme with food bank	Food and Health Development Worker for this Community food and health project	Supports vulnerable people living in food poverty. Promotes healthy eating via cookery classes	Lothian	Disadvantaged groups in deprived areas of city—mainly serves families with young children
